# EGFR T790M detection and osimertinib treatment response evaluation by liquid biopsy in lung adenocarcinoma patients with acquired resistance to first generation EGFR tyrosine kinase inhibitors

**DOI:** 10.1186/s13000-018-0728-6

**Published:** 2018-08-13

**Authors:** Chenguang Li, Rui Jia, Hailin Liu, Bin Zhang, Changli Wang

**Affiliations:** 1 Department of Lung Cancer, Tianjin Medical University Cancer Institute and Hospital, Tianjin, China; 2 National Clinical Research Center for Cancer, Tianjin, China; 3 Key Laboratory of Cancer Prevention and Therapy, Tianjin, China; 4 Tianjin Lung Cancer Center, Tianjin, China

**Keywords:** NSCLC, ctDNA, Epidermal growth factor receptor, T790M

## Abstract

**Background:**

Lung adenocarcinoma with EGFR activating mutations will inevitably acquire resistance to first generation TKIs. Acquired EGFR T790M mutation causes about 50% of these resistance cases. Droplet digital PCR (ddPCR) and cobas enables quantification of T790M mutation. Whether these methods can predict clinical response of osimertinib treatment is unknown.

**Methods:**

Tumor and blood samples from 69 stage IIIB-IV NSCLC patients acquired resistance to EGFR-TKI were collected. Cobas® Mutation Test v2 kit was used to detect EGFR mutations in FFPE or plasma samples. Plasma T790M mutation of both osimertinib naïve and treated patients were quantified by Droplet digital PCR (ddPCR).

**Results:**

T790M mutation rate detected by FFPE tissue cobas, plasma ctDNA cobas and plasma ctDNA ddPCR test were 54.5, 21.3 and 30.4% respectively. The T790M positive rate was 52.2% considering all testing methods. The objective response rate (ORR) was 60.9% in 23 patients received osimertinib treatment. Quantification of T790M after treatment decreased to very low level, but no association was observed between clinical response and T790M mutation level decrease.

**Conclusion:**

ddPCR is more sensitive in plama ctDNA testing and should be performed even in tumor tissue T790M test negative cases. EGFR T790M mutation level is not associated with clinical response after osimertinib treatment.

## ᅟ

Part result of this study was published as abstract and poster presentation in IASLC 18th World Conference on Lung Cancer [1].

## Background

Lung cancer remains the leading cause of cancer-related death worldwide [2]. Though epidermal growth factor receptor tyrosine kinase inhibitors (EGFR-TKIs) have significantly improved outcome of EGFR mutation positive advanced non-small cell lung cancer (NSCLC), most of these patients develop resistance to first generation EGFR TKIs after a median of 10–12 months, in which about 50–60% of these tumors harbor EGFR T790M resistance mutation [3–5]. Osimertinib is an oral, irreversible EGFR-TKI that is selective for both EGFR activating and T790M resistance mutations [6]. In AURA3 study, osimertinib showed significant advantage against platinum therapy plus pemetrexed in both progression-free survival and objective response rate [7].

To obtain enough tumor tissue in patients experienced disease progression is challenging, especially in patients receiving first generation EGFR-TKI treatment. In other cases, tumor biopsy tissue may not reflect the overall tumor mutational features due to high tumor burden and heterogeneity. The use of circulating tumor DNA (ctDNA) to detect genetic alterations by various platforms known as liquid biopsy has provided a non-invasive option [8]. The current study focuses on the efficacy of T790M detection by different platforms, as well as T790M level response after receiving osimertinib treatment.

## Methods

### Patients

Plasma and tumor tissue samples were collected from patients screened or enrolled in the ongoing ASTRIS study. ASTRIS (D5160C00022) is open label, multinational, multicenter, real world treatment study of single agent osimertinib for patients with advanced/metastatic EGFR T790M mutation positive NSCLC who have received prior therapy with an first generation EGFR-TKI. Patients were required to have the diagnosis of EGFR activating mutation positive NSCLC. Drug resistance was defined as disease progression after first generation EGFR-TKI (gefetinib, elortinib or ecotinib) treatment, which was evaluated by RECIST 1.1 criteria. EGFR T790M positive patients received osimertinib 80 mg Qd until disease progression evaluated by investigators.

### Tumor tissue and plasma sample collection and mutational analysis

Tumor tissue or plasma samples were collected from patients screened or enrolled in ASTRIS study, following progression on a previous EGFR-TKI, but prior to dosing with osimertinib. CT-guided needle lung biopsy or Supraclavicular lymph node biopsy samples were snap frozen and stored at − 80 °C. A total of 8 ml of Plasma sample from each patient were assigned for cobas and ddPCR testing, and 4 ml of plasma from the time point of first evaluation (6 weeks) after osimertinib treatment were evaluated by ddPCR. ctDNA isolation followed standard protocols. Cobas® Mutation Test v2 kit was used to detect both EGFR activating mutation and T790M in FFPE or plasma samples. The Cobas is an allele-specific polymerase chain reaction assay to detect known common EGFR mutations. Analysis was confirmed by negative and positive controls. The PCR reactions were run on the cobas®z 480 analyzer with EGFR Blood Analysis Package Software. A semiquantitative index (SQI) was generated to reflect the proportion of mutated and wild-type copies of the EGFR gene. The SQI was derived from a dilution series with known copy numbers of mutated EGFR and a fixed amount of wild-type EGFR, with the wildtype DNA copy as internal control. Bio-rad PrimePCR™ ddPCR™ Mutation Assay kit were used to detect T790M in plsma ctDNA. The RNase P Copy Number Reference Assay (Life Technologies, Carlsbad, California) was used to determine the total amount of DNA in plasma samples. A total of 3000–3500 genome equivalents were analyzed per reaction with a sensitivity between 0.1–0.5%.

### Statistical analysis

Associations between mutations and clinical and biological characteristics were analyzed by *χ*^*2*^ or Fisher’s exact test. All data were analyzed using the Statistical Package for the Social Sciences Version 16.0 Software (SPSS Inc., Chicago, IL). The two-sided significance level was set at *p* < 0.05.

## Results

### Study design and patient characteristics

**Fig. 1:**
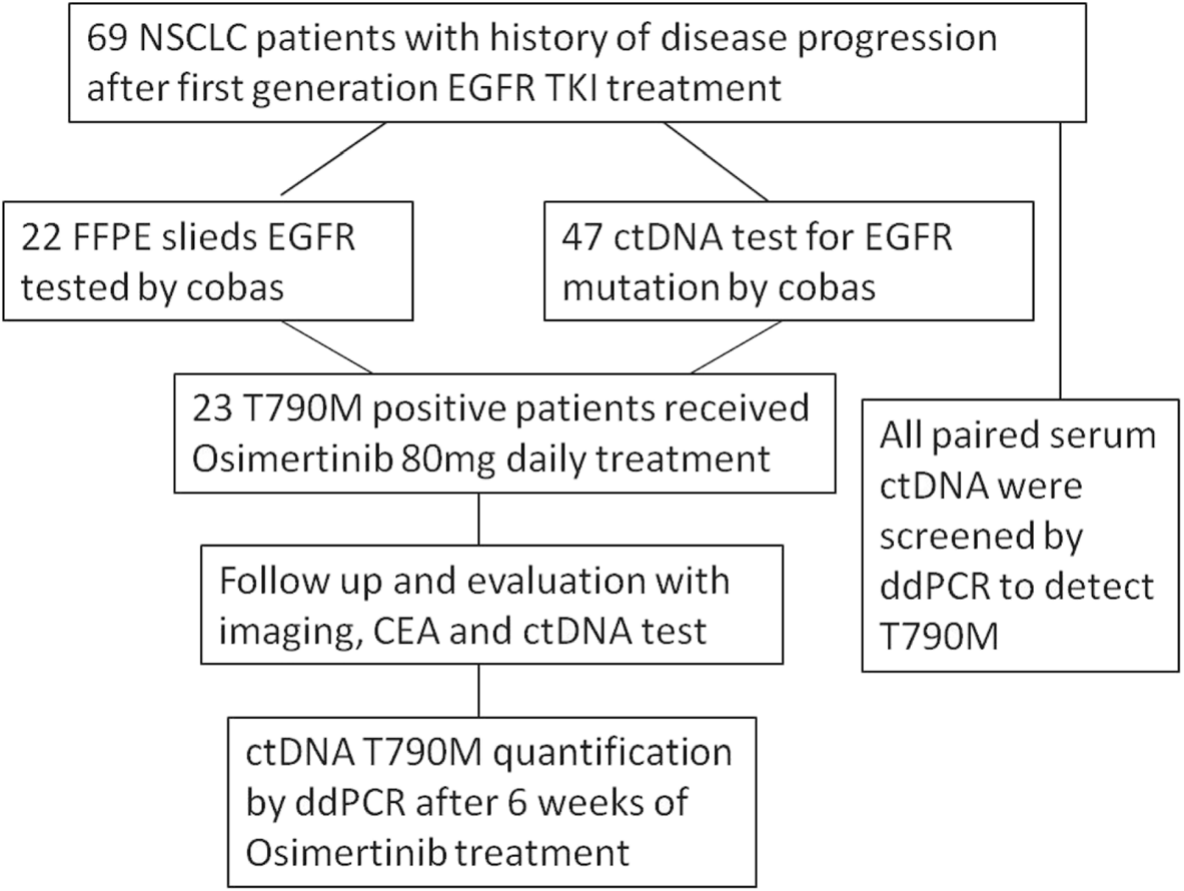
Flowchart of the study design

The flowchart of study design was shown in Fig. 1. Among the 69 screened NSCLC patients, there were 23 men and 46 women. There were 22 smokers and 47 non-smokers, accounting for 31.9 and 68.1%, respectively. There were 67 adenocarcinoma and 2 Adenosquamous carcinoma. The number of patients in stages IIIB, IVA and IVB was 20, 21, and 28, respectively. The numbers of M1a, M1b and M1c patients were 19, 4 and 26. The detailed information is listed in Table 1.

**Table 1:** Demographics and clinicopathologic features of 69 NSCLC patients screened for T790 M mutation

Variables	No. of patients (%)
Gender	
Male	23(33.3)
Female	46(66.7)
Age	
≤ 40	5 (7.2)
> 40	64(92.8)
ECOG performance status	
0–1	58(84.1)
2	11(15.9)
Histologic subtype	
Adenocarcinoma	67(97.1)
Adenosquamous carcinoma	2 (2.9)
Smoking status	
Smoker	22(31.9)
Non-smoker	47(68.1)
TNM Stage	
IIIB	20(29.0)
IVA	21(30.4)
IVB	28(40.6)
M Stage	
M1a	19(38.8)
M1b	4 (8.2)
M1c	26(53.0)
First generation EGFR TKIs	
Gefitinib	22(31.8)
Elortinib	15(21.7)
Ecotinib	31(44.9)
Two or more TKI drugs	1 (1.4)
EGFR mutation detection	
FFPE slieds by cobas	22
cfDNA by cobas	47
T790 M ddPCR assay	69
Total	69

### Detection of T790M mutation by cobas® and ddPCR platform

**Fig. 2:**
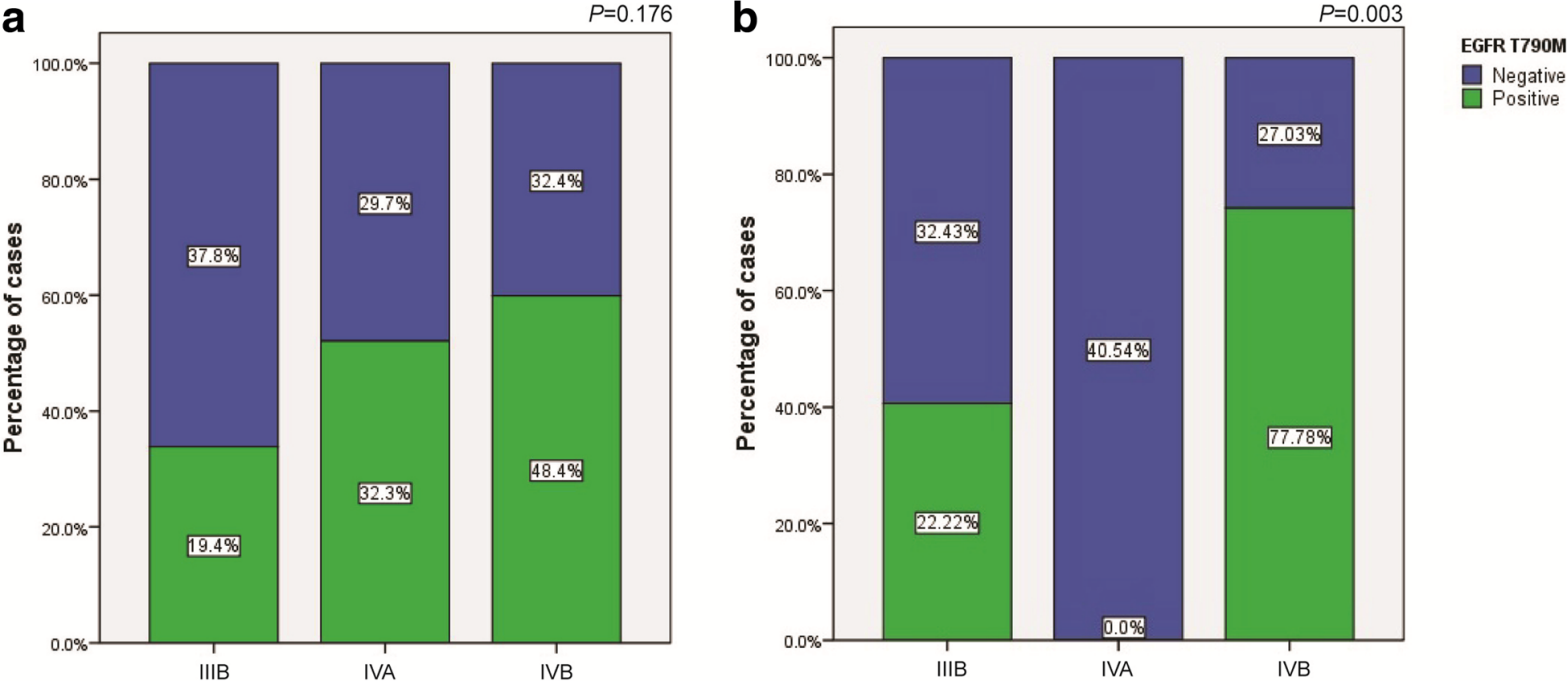
Distribution of T790 M positive and negative cases in stage IIIB-IVB NSCLC patients with acquired resistance to first generation EGFR TKIs. **a**.ctDNA T790 M mutation detected by ddPCR; **b**.ctDNA T790 M mutation detected by Cobas

The T790M mutation rate of FFPE tissue cobas, plasma cobas and plasma ddPCR were 54.5, 21.3 and 30.4% respectively. The T790M positive rate was 52.2% considering all testing methods. T790M mutation was detected by ddPCR in all of the plasma cobas positive cases. However, in 10 tumor tissue biopsy cobas negative cases, 3 were positive by plasma ctDNA ddPCR. The proportion of T790M positive cases detected by ddPCR in stage IIIB, IVA and IVB patients were 30, 47.6 and 57.1%, respectively (*P* = 0.176) (Fig. 2a). However, T790M positive defined by cobas plasma ctDNA test were significantly different in stage IIIB, IVA and IVB (Fig. 2b) or M1a, M2b and M1c patients (*P* = 0.003, 0.004), suggesting the advantages of ddPCR test in any stages of disease. The T790M quantification by ddPCR significantly rises in stage IIIB, IVA and IVB cases (*P* = 0.033), while no such trend was observed in different M1a, M2b and M1c cases (*P* = 0.178). No association was observed between T790M status and duration of first generation EGFR TKI treatment (Table 2).

**Table 2:** Association between EGFR T790 M mutation and clinical features in patients with disease progression after receiving 1st generation EGFR TKI treatment

		EGFR T790M detected by any method			EGFR T790M detected by plama ctDNA ddPCR			T790M detected by cobas plama ctDNA test			Cobas Activating Mutation SQI		Cobas T790M SQI		ddPCR T790M quantitative	
Characteristics	Total	Mutant	Wild type	*P*	Mutant	Wild type	*P*	Mutant	Wild type	*P*	Mean	*P*	Mean	*P*	Mean	*P*
Gender																
Male	23	12	11	1	11	12	0.864	2	12	0.7	10.24	0.859	9.81	0.101	0.52	0.307
Female	46	24	22		21	25		8	25		10.55		8.17		1.4	
Smoking status																
Smoker	22	11	11	0.805	10	12	0.916	3	11	1	10.59	0.897	9.8	0.472	1.63	0.384
Non-smoker	47	25	22		22	25		7	26		10.37		7.94		0.86	
Clinical Stage																
IIIB	20	9	11	0.495	6	14	0.176	2	12	**0.003**	9.24	0.444	7.8	0.77	0.29	**0.033**
IVA	21	10	11		10	11		0	15		10.03		NA		0.2	
IVB	28	17	11		16	12		8	10		11.51		8.68		2.37	
M Stage																
M1a	19	9	10	0.627	9	10	0.827	0	15	**0.004**	10.03	0.541	NA	NA	0.21	0.178
M1b	4	2	2		2	2		0	1		NA		NA		1.07	
M1c	26	16	10		15	11		8	9		11.51		8.5		2.4	
Time of EGFR-TKI treatment																
≤ 6 months	14	6	8	0.733	6	8	0.952	1	6	0.621	10.5	0.082	8.99	0.203	0.83	0.472
6–12 months	26	14	12		12	14		5	12		12.71		10.32		1.75	
>12 months	29	16	13		14	15		4	19		8.86		6.11		0.67	

*ddPCR* Droplet Digital PCR, *SQI* Semi-Quantitative Index, Bold numbers showing statisticaly significant results

### Evaluation of plasma T790M level during osimertinib treatment by ddPCR

**Fig. 3:**
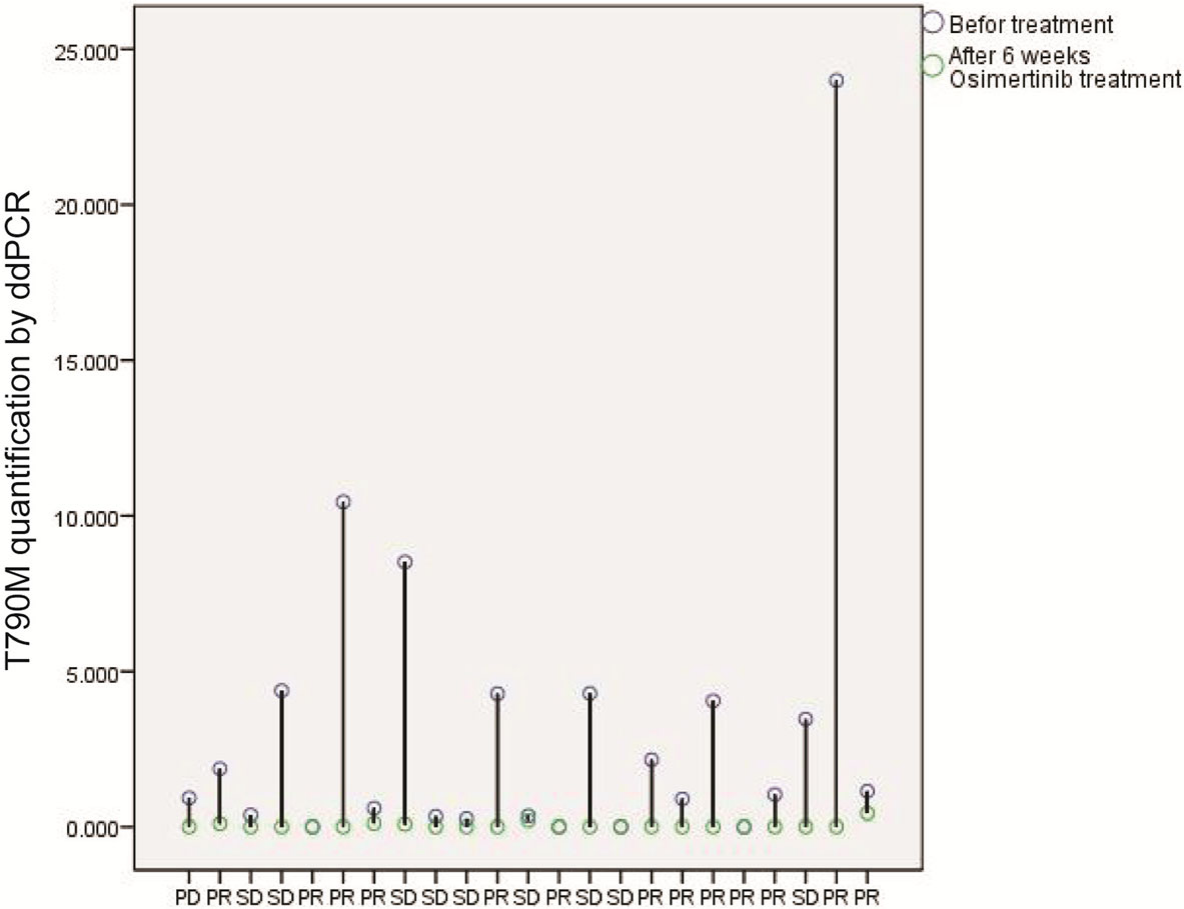
ctDNA T790 M quantification by ddPCR before and after osimertinib treatment. PD, Progressive Disease; PR, Partial Response; SD, Stable Disease

In 23 patients received osimertinib treatment, the OOR was 60.9%. There were 14 patients evaluated as partial response (PR) and 8 were stable disease (SD), 1 patient experienced PR of liver metastasis tumor but progression of primary lung lesion. Quantification of T790M after 6 weeks of treatment decreased to very low level, while no association was observed between response status and T790M mutation level decrease (Fig. 3).

## Discussion

The aim of this study was to evaluate different T790M detecting methods in advanced NSCLC patients who experienced disease progression after receiving EGFR TKI treatment, as well as T790M quantification after osimertinib treatment. Two quantification methods were tested on a cohort of 69 patients enrolled in this single center as part of the multicenter real-world ASTRIS study. These patients represent outline features of Chinese patients who experienced disease progression after gefetinib, elortinib or ecotinib treatment. Plasma samples were collected at screening and 6 weeks after receiving osimertinib treatment. The overall T790M positive rate was 52.2% considering all testing methods, the ORR of T790M positive patients receiving osimertinib treatment was 60.9%. These data were similar compared with published data [7, 9–11].

Our analysis revealed a rising trend of T790M positive rates detected by ddPCR in stage IIIB, IVA and IVB patients. In plasma ctDNA samples tested by cobas, T790M positive rate was significantly higher in stage IVB than stage IIIB and IVA, M1c than M1a and M1b patients. On one side, more advanced stage represents significantly higher tumor burden, in which case tumor shed more ctDNA to the bloodstream [12, 13]. On the other side, these results indicate that the cobas test is less capable of detecting relatively earlier stage cases.

In all of the plasma ctDNA cobas test T790M positive samples, ddPCR test also yielded positive results. Even in 10 tumor tissue test negative cases, 3 were positive defined by plasma ctDNA ddPCR test. These results suggest that plasma ctDNA ddPCR test is more sensitive and should be used as primary choice in managing patients with resistance to first line EGFR TKIs. The reason of inconsistency between tumor tissue test and ddPCR test is probably due to tumor heterogeneity in primary and metastatic tumors, as well as intratumor heterogeneity. These facts suggests co-existing of multiple resistant clones or single clone harboring multiple resistance mechanism [14, 15]. Plasma ctDNA ddPCR test should be routinely performed in such cases considering its noninvasive and low cost feature.

Most of patients showed a PR or SD status after the evaluation of 6 weeks after receiving osimertinib treatment, generating an ORR of 60.9%. We also compared the ctDNA T790M level in pre and post osimertinib treatment plasma samples. Though all plasma ctDNA T790M decreased to very low level, no association was observed with radiographic response. Previous studies dynamically monitored EGFR mutation status using plasma samples by ddPCR to evaluate response to first generation EGFR TKIs [16]. Another study quantified plasma T790M level in two cases of patients who received osimertinib treatment [17]. In the present study, we have quantified T790M level in relatively large number of samples. Though quantification of plasma ctDNA T790M didn’t predict response in short term, dynamic monitoring may indicate disease progression in the long run.

## Conclusion

In conclusion, our data suggest that ddPCR is more sensitive in plama ctDNA testing and should be performed even in tumor tissue T790M test negative cases. Osimertinib significantly decreased plasma T790M level, but no association was observed between plasma ctDNA T790M level decrease and clinical response.
